# Circadian Rhythm Genes and Their Association with Sleep and Sleep Restriction

**DOI:** 10.3390/ijms251910445

**Published:** 2024-09-27

**Authors:** Marcin Sochal, Marta Ditmer, Aleksandra Tarasiuk-Zawadzka, Agata Binienda, Szymon Turkiewicz, Adam Wysokiński, Filip Franciszek Karuga, Piotr Białasiewicz, Jakub Fichna, Agata Gabryelska

**Affiliations:** 1 Department of Sleep Medicine and Metabolic Disorders, Medical University of Lodz, 92-215 Lodz, Poland; marta.insmk@gmail.com (M.D.); turkiewiczszymon99@gmail.com (S.T.); filip.karuga@stud.umed.lodz.pl (F.F.K.); piotr.bialasiewicz@umed.lodz.pl (P.B.); agata.gabryelska@gmail.com (A.G.); 2 Department of Biochemistry, Medical University of Lodz, 90-419 Lodz, Poland; tarasiuk.aleksandra@gmail.com (A.T.-Z.); agata.binienda@stud.umed.lodz.pl (A.B.); jakub.fichna@umed.lodz.pl (J.F.); 3 Department of Old Age Psychiatry and Psychotic Disorders, Medical University of Lodz, 90-419 Lodz, Poland; adam.wysokinski@umed.lodz.pl

**Keywords:** sleep deprivation, clock genes, circadian rhythm

## Abstract

Deprivation of sleep (DS) and its effects on circadian rhythm gene expression are not well understood despite their influence on various physiological and psychological processes. This study aimed to elucidate the changes in the expression of circadian rhythm genes following a night of sleep and DS. Their correlation with sleep architecture and physical activity was also examined. The study included 81 participants who underwent polysomnography (PSG) and DS with actigraphy. Blood samples were collected after PSG and DS. Expression levels of brain and muscle ARNT-like 1 (BMAL1), circadian locomotor output cycles kaput (CLOCK), neuronal PAS domain protein 2 (NPAS2), period 1 (PER1), cryptochrome 1 (CRY1) and nuclear receptor subfamily 1 group D member 1 (NR1D1) were analyzed using qRT-PCR. DS decreased the expression of CLOCK and BMAL1 while increasing PER1. PER1 expression correlated positively with total sleep time and non-rapid-eye-movement (NREM) sleep duration and negatively with sleep latency, alpha, beta and delta waves in the O1A2 lead. Physical activity during DS showed positive correlations with CLOCK, BMAL1, and CRY1. The findings highlight the role of PER1 in modulating sleep patterns, suggesting potential targets for managing sleep-related disorders. Further research is essential to deepen the understanding of these relationships and their implications.

## 1. Introduction

Deprivation of sleep (DS) refers to a deliberate restriction of sleep, which can result from external factors like work demands or noise or internal factors such as pain [1]. This condition differs from insomnia, where individuals struggle to fall asleep despite suitable conditions. Over recent decades, the relationship between sleep disturbances and the circadian rhythm has garnered significant interest, though the molecular mechanisms underpinning this association are not fully understood [2].

The circadian rhythm orchestrates various biological processes, including cellular metabolism and the sleep/wake cycle, through a network of clock genes. These genes, such as brain and muscle ARNT-like 1 (BMAL1), circadian locomotor output cycles kaput (CLOCK), neuronal PAS domain protein 2 (NPAS2), and nuclear receptor subfamily 1 group D member 1 (NR1D1), are widely expressed across tissues and might function to some extent independently of the central master clock in the suprachiasmatic nucleus [3]. A key mechanism driving the circadian rhythm involves the interaction between the CLOCK/BMAL1 heterodimer and the period circadian protein homolog (PER) and cryptochrome (CRY) proteins, facilitated by the transcriptional regulator E-box [3]. In mammals, the CLOCK/BMAL1 complex activates PER and CRY transcription by binding to the E-box, after which PER and CRY proteins inhibit their own transcription [3]. The NR1D1 protein further modulates this process by repressing BMAL1 and CLOCK gene expression, while NPAS2 can dimerize with BMAL1, functioning as a substitute for CLOCK [3].

Even though lack of sleep is known to have a range of negative health consequences, a single night of DS has been studied as a therapeutic intervention in the treatment of depression [4]. Research on sleep deprivation is particularly challenging due to the absence of a standardized DS protocol and the demanding nature of these studies on participants; thus, the results tend to vary between the studies vastly. Despite extensive research, the molecular bases of these changes are still poorly understood, and the individual factors influencing patient responses remain unclear. Identifying these factors is crucial, as they could lead to more personalized and effective treatments.

Clock genes might be the link between sleep (or, in consequence, a lack thereof) and mental health. Their disturbances have long been connected to psychiatric disorders, such as depression, anxiety, bipolar disorder, or seasonal affective disorder, all of which might manifest in the form of sleep disturbances. Mutations of clock genes were observed to affect animal behavior. Clock-mutant mice showed reduced anxiety, whereas animals with decreased PER1 and PER2 expression were more likely to exhibit fearful behavior [5,6]. Lamont et al. suggested that both electroconvulsive therapy and antidepressant medications might affect the activity of the GSK3 enzyme, which could act as a modulator of the circadian rhythm, among others, via phosphorylation of the PER2 and NR1D1 [7]. Furthermore, several polymorphisms of PER2, NPAS2, and BMAL1 were associated with seasonal affective disorder [7].

Physical activity may also modulate the effects of sleep deprivation on mood, potentially linked to its influence on neurotrophins and clock genes. While research confirms that exercise boosts the expression of these genes, the specific impacts under sleep-deprived conditions remain less explored [8,9].

Basal expression of clock genes influences sleep-related molecular aspects, impacting both subjective sleep quality and EEG parameters [10]. EEG, essential for diagnosing sleep and neurological disorders, records brain activity via scalp electrodes, revealing unique patterns across different sleep stages [11]. These patterns include various wave activities in REM and non-REM phases, with EEG power and slow-wave activity serving as critical indicators of sleep physiology [11]. Studies on the connection between clock genes and sleep deprivation are sparse. Research performed on animals shows that DS affects the forebrain the most, compared to other regions, by increasing the expression of the PER1 gene and delta power in EEG; however, the authors did not try to correlate those variables [12,13].

As for humans, studies involving biopsies from the central nervous system are, for obvious reasons, limited; however, there are some clues connecting brain function, clock genes, and sleep. Individuals homozygous for the longer PER allele (PER3^5/5^) exhibited significant changes in sleep architecture, affecting various indicators of sleep homeostasis [14]. This included an increase in slow-wave sleep (SWS) and EEG slow-wave activity during non-rapid eye movement (non-REM) sleep, as well as heightened theta and alpha activity during both wakefulness and REM sleep, compared to those with the PER3^4/4^ genotype [14]. Additionally, the PER3^5/5^ allele predisposes individuals to decreased cognitive performance after DS [14]. Despite the partial understanding of the interactions between clock genes and sleep, more studies, especially those involving human DS models, could further elucidate the intricacies of this relationship, including the negative and positive aspects of DS.

This study aims to explore changes in the expression of circadian rhythm genes (CLOCK, BMAL1, CRY1, PER1, NR1D1) following a full night’s sleep and sleep deprivation. Additionally, it investigates factors that could affect the expressions of these genes and determine their association with selected polysomnography parameters.

## 2. Results

A total of 81 participants were included in the study. In seven of them, it was not possible to measure the expression of any of the studied genes. In the remaining participants, logarithmic relative expression was not determined for all genes and at all time points (the number of participants in whom individual genes were measured is given below for each gene). The characteristics of the studied population are presented in Table 1.

Before PSG, in comparison with female subjects, male participants exhibited decreased expression of NR1D1 (−4.4 IQR: −4.8–(−3.6) vs. −3.8 IQR: −4.1–(−3.1), *p* = 0.028), CRY1 (−4.3 IQR: −5.1–(−3.6) vs. −3.8 IQR: −4.2–(−3.2), *p* = 0.013) and CLOCK (−4.1 IQR: −4.6–(−3.2) vs. −3.6 IQR: −3.9–(−2.9), *p* = 0.020); no such differences were detected at other timepoints. A decrease in relative expression was observed in all studied genes after a night’s sleep, measured between the evening and the morning (Table 2).

After sleep deprivation, no significant changes in the expression of CLOCK, BMAL1, and PER1 genes were observed between evening and morning collection. A decrease in relative expression was observed for the genes CRY1, NR1D1, and NPAS2.

A comparison of morning blood draws after a night’s sleep and after sleep deprivation showed that the expression of CLOCK and BMAL1 genes was significantly lower after sleep deprivation, while PER1 expression increased. No such associations were observed for the genes CRY1, NR1D1, and NPAS2 (Table 2).

Morning PER1 levels measured after a night’s sleep positively correlated with TST and NREM duration and negatively with sleep latency. No such correlation was observed for REM duration (Table 3). The remaining studied genes did not correlate with PSG parameters.

Expression of CLOCK, BMAL1, and CRY1 positively correlated with total activity during sleep deprivation. No such correlation was observed for the other studied genes.

No correlations were observed between brain waves and circadian rhythm genes in the REM sleep stage. In the NREM stage, the PER1 gene negatively correlated with the power of alpha, beta, and delta waves (but not theta) in the occipital leads but not in the other studied leads (Table 4).

Out of the remaining genes, only CLOCK positively correlated with the power of the delta wave in the F4A1 lead (R = 0.34, *p* = 0.036). NR1D1 and NPAS2 negatively correlated with the power of delta waves measured in the O1A2 lead (R = −0.34, *p* = 0.036; R = −0.38, *p* = 0.039, respectively).

Multiple regression, using the progressive step method, revealed that PER1 expression was significantly affected by delta power waves but not by alpha and beta power waves in the O1A2 lead. The obtained model explained the 18% variability of PER1 (R^2^ = 0.18, b = 7.84, *p* = 0.021).

## 3. Discussion

The effects of sleep deprivation on humans have been extensively studied since the late 19th century, with early research indicating that acute sleep loss can alter mood and cognitive abilities and potentially have an antidepressant effect, although findings are inconsistent across studies [15]. Previous studies on the impact of sleep deprivation on mental health have yielded ambiguous results, and these differences may be explained by molecular mechanisms. Circadian rhythm genes in the context of sleep deprivation have been investigated, but these studies typically utilized animal models or very small human cohorts, which significantly limited the conclusions that could be drawn.

This is the first study on a human model corroborating the relationship between the PER1 gene and sleep. PER1 showed a negative correlation with several sleep parameters: TST, REM/NREM duration, and sleep latency. EEG recordings revealed that PER1 negatively correlated with alpha, beta, and delta waves in the O1A2 lead during the NREM stage. Further analysis indicated a significant link between PER1 and delta power, a marker for the biological need for sleep. In the same O1A2 lead, NR1D1 and NPAS2 also showed a negative correlation with delta power. This connection between homeostatic sleep regulation and PER genes seems to be further corroborated by other studies. Kopp et al., in their study on mice, showed that PER-knockouts still had a physiological response to DS, i.e., delta power increased primarily in the area of the frontal cortex, albeit of a lesser magnitude than wild-type controls [12]. Other studies confirmed that DS seems to affect the frontal region the most [13]. Franken et al. noted that after DS, the increase in the expression of the PER1 gene was primarily seen within the frontal cortex and the cerebellum [13]. In contrast to the previously mentioned studies on rebound sleep, here, during PSG, most changes in the expression of PER1 showed a connection to EEG only in the occipital region [13]. Additionally, we noted a negative correlation between CLOCK expression and delta power in the F4A1 lead. Those findings suggest that rebound sleep, apart from changes in EEG delta power, might possess other distinctive characteristics, discerning it from regular sleep.

In the present study, it was observed that DS resulted in decreased expression of BMAL1, CLOCK, and CRY1, whereas the expression of PER1 increased. Most animal studies to date have focused on the expression of these genes within the central nervous system; it is also known that DS can increase the expression of period genes in the brains of animals [16]. It is crucial to note that these changes can be tissue-specific; for example, DS increased the expression of NPAS2 in the forebrain but not in the cortex [17,18,19]. Wisor et al. reported that the expression of BMAL1 and CLOCK could be upregulated following sleep loss, although these effects varied by strain [17]. Those findings warrant the search for a correlation between the expression of clock genes and EEG in leads analyzing different parts of the brain, which was performed in the present study. Due to safety considerations, the investigation of clock gene expression in human brain tissue is significantly restricted. Concurrently, it is known that every cell in the body possesses its own biological clock in the form of clock genes, which justifies the study of their expression in peripheral tissues [20,21]. Gene expression analysis, especially for circadian clock genes, is usually performed using peripheral blood mononuclear cells (PBMCs) instead of whole blood. This is due to the fact that granulocytes, which make up the majority of white blood cells, are released into the bloodstream in a circadian manner, potentially skewing the results [22]. The so-called “buffy coat”, obtained via centrifugation of the whole blood, consists mostly of PBMCs, allowing for proper evaluation of mRNA production.

Ackermann et al. reported reduced BMAL1 and CRY1 expression in PBMCs, with no significant changes in CLOCK gene expression, possibly due to the small study size (*n* = 12, all male) [23]. Kavčič et al. also studied PBMCs and found diurnal variations in PER2 and BMAL1 expression, with a loss of oscillatory patterns for PER2 under sleep deprivation conditions but not for BMAL1, contrasting with Ackermann et al.’s findings where circadian oscillations in PER2 expression were absent even in normal conditions [23,24]. They also observed only a slight delay (about one hour) in BMAL1 peak expression [24]. In comparison, James et al. found that DS shifted the peak expression of BMAL1 [21]. PER1 and PER2, on the other hand, maintained their rhythmicity [21]. This study, however, faced limitations similar to others, including a small sample size (*n* = 6, including 4 males and 2 females) [21]. As for sex differences, in the present study, it was noted that males had lower expression of CLOCK, CRY1, and NR1D1 than female participants before the PSG but not after PSG or DS. Several previous studies noted that sexual dimorphism might manifest itself also in this domain [25,26,27]. Sex hormones are also well known to mutually interact with circadian clock genes [28]. The exact mechanisms behind the discussed relationship are outside the scope of this work. Since no differences were detected after a full night’s sleep or DS, it seems that this characteristic did not influence the results of the present study.

The study did not focus on mood changes following DS, but research suggests that clock genes play a pivotal role in the type of mood response to DS, possibly explaining its antidepressant effects [29,30]. Interestingly, the antipsychotic medication quetiapine, used to treat major depression, has also been shown to increase PER1 expression in the amygdala [31]. In another study involving animals, mice lacking PER1 and PER2 expression exhibited increased anxiety compared to wild-type controls [5]. The administration of fluoxetine was found to normalize the expression of these PER genes, further implicating their role in the pathophysiology of psychiatric disorders [5]. What is worth noting is that both PER genes are considered to be immediate-early genes whose transcription is controlled by various environmental and systemic cues, including light [32,33]. In the present study, participants did not have a constant routine, and light exposure was largely self-regulated. It is possible that the apparent PER photosensitivity affected its changes during DS.

Lavabratt et al. reported increased CRY2 expression following DS in healthy individuals but not in those with bipolar disorder during depressive episodes [34]. In a study by Bunney et al., responders to DS therapy exhibited significant increases in the expression of circadian clock genes (RORA, DEC2, and PER1) following DS, whereas non-responders showed decreases [29]. This may suggest a reset of the circadian clock, which normalizes during recovery sleep. Chronotherapies and other interventions targeting circadian rhythms offer promising avenues for maintaining these antidepressant effects. In the present study, candidates with severe mental health issues were excluded.

To the best of our knowledge, this is the first study investigating the relationship between clock genes and physical activity under the conditions of acute sleep deprivation. In the previous study, we demonstrated that physical activity results might influence the expression of neurotrophins during DS, which could impact mood response to acute sleep loss [8]. Here, actigraphy outcomes positively correlated with the expression of major clock genes, namely BMAL1, CLOCK, and CRY1, in the morning. This is in line with other studies, which show that both resistance and aerobic training might increase the expression of clock genes [35]. An interesting aspect of the relationship between circadian rhythm and physical activity is that it might affect the expression of clock genes differently depending on the time of the day. Tanaka et al., in their study, demonstrated that CRY1 expression was elevated after morning training, whereas BMAL1 was elevated after both morning and afternoon exercises. CLOCK and PER genes were not affected [36]. An important limitation of the study was a small (*n* = 11), male-only study group [36]. Cedernaes et al. explored the expression of circadian clock genes in adipose tissue and skeletal muscles during DS and observed no significant changes in gene expression, though DS appeared to enhance the methylation of the CRY1 promoter and two regions associated with increased PER1 expression [37]. In contrast, in skeletal muscle, DS reduced the expression of BMAL1 and CRY1. However, the study group was small (*n* = 15 males) [37]. Those results could be extrapolated to predict gene expression in leukocytes, as numerous studies have demonstrated similarities in gene expression between skeletal muscles and PBMCs [38,39]. Nevertheless, physical activity might induce certain tissue-specific changes. For instance, Gjevestad et al. reported that strength training increased the expression of pro-inflammatory mediators in both skeletal muscles and PBMCs [40]. However, several cytokines, including interleukin-6, chemokine (C-C motif) ligand 2, and tumor necrosis factor, were elevated exclusively in muscle biopsies [40].

The present study distinguishes itself from other projects involving sleep deprivation in several ways. First, it has a large number of participants, which allowed for the detection of changes that could have been otherwise missed. Second, changes in clock gene expressions were correlated with EEG parameters obtained during PSG conducted in a sleep lab, which gives the foundation for future studies analyzing mechanisms regulating circadian rhythm in different parts of the brain. Third, the protocol utilized an innovative approach to DS, allowing the subjects to spend the night as they pleased while monitoring physical activity, which has a well-documented influence on circadian rhythm with an actigraph.

While this study has made strides in understanding the peripheral manifestations of circadian rhythm disruptions due to sleep deprivation, it has several limitations. Assessment of data regarding mood, cognitive abilities, etc., was also omitted. Participants enrolled in the study were generally healthy, which does not allow for the extrapolation of the results onto depressed subjects in future projects. Expression of the clock genes was analyzed only from venous blood; the protocol did not include obtaining biopsies of skeletal muscles or central nervous system for obvious reasons. PSG was conducted only once; thus, its results could be skewed by the ‘first night effect’. An actigraph might miss short naps or mistake periods of inactivity for sleep, especially since DS was not conducted in a controlled environment such as a sleep laboratory. In the same vein, since constant routine was not included in the protocol, observed changes in clock gene expressions could be attributed to the light/dark cycle, being more of a diurnal nature rather than circadian.

To summarize, sleep deprivation induced a decrease in the expressions of CLOCK and BMAL1, whereas PER1 expression increased. Importantly, morning PER1 levels positively correlated with total sleep time and NREM duration but negatively with sleep latency, suggesting its influence on sleep architecture. Additionally, in the NREM stage, PER1 negatively correlated with alpha, beta, and delta EEG wave powers in the O1A2 lead, providing insights into the interplay between gene expression and brain activity during sleep. Conversely, expressions of CLOCK, BMAL1, and CRY1 correlated positively with total activity during sleep deprivation. These results highlight the nuanced impacts of sleep and sleep deprivation on the dynamics of circadian gene expression, suggesting potential biological pathways through which sleep quality, brain activity, and physical activity can affect circadian regulation.

Future studies should continue to explore the complex relationship between sleep deprivation, circadian rhythms, and mood regulation. The study reinforces the complex relationship between sleep deprivation, physical activity, circadian rhythm disturbances, and their broader physiological and psychological implications. Continued exploration in this field is essential to fully understand and harness the circadian components in managing sleep and mood disorders effectively.

## 4. Materials and Methods

### 4.1. Study Group

One hundred and thirteen participants were enlisted to partake in the investigation. Among them, 32 individuals discontinued the protocol. Subsequently, data from the remaining 81 subjects were subjected to analysis.

The study protocol received approval from The Bioethical Committee of the Medical University of Lodz (reference number: RNN/302/20/KE). Specific inclusion criteria were enforced, including obtaining informed consent for participation in both phases of the study (PSG and DS) through the completion of the relevant documentation provided by the Department of Sleep Medicine and Metabolic Disorders, adherence to the study protocol, age range between 18 and 35 years, and a body mass index (BMI) falling within the range of 20 to 30 kg/m^2^.

The criteria for exclusion encompassed pregnancy or breastfeeding, chronic ailments (including inflammatory, endocrine, cardiopulmonary, or renal insufficiency), undergoing radio or chemotherapy, presence of neoplasms aside from basal-cell carcinoma, recent surgery within the past 6 months, substance dependence, existence of sleep disorders, and undertaking intercontinental flights within 2 weeks prior to qualification.

A diagnosis of mental disorders was excluded based on a medical history and questionnaires (obsessive-compulsive disorder—Yale-Brown Obsessive-Compulsive Scale and bipolar disorder—Mood Disorder Questionnaire).

### 4.2. Protocol

The research procedure comprised two phases: polysomnography (PSG) and deprivation of sleep (DS). Participants were admitted to the Department of Sleep Medicine and Metabolic Disorders at the Medical University of Lodz on the scheduled evening for the standard PSG assessment. A physician conducted a general physical examination and gathered medical history after that. The PSG session was scheduled for a duration of 9 h, spanning from 10 P.M. to 7 A.M.

The PSG findings were assessed according to the guidelines set forth by the American Academy of Sleep Medicine, with an epoch length of 30 s [41].

The PSG was conducted using Alice 4, Phillips-Respironics (Monroeville, PA, USA). The examination allowed for the collection of the following parameters: electromyography/oculography/encephalography/cardiography, SpO_2_, respiratory effort, airflow, and body position. A single researcher performed the interpretation of PSG recordings to ensure consistency in results.

EEG channels exhibiting electrode impedance exceeding 5 KΩ were excluded from the analysis. EEG data were sampled with a sampling rate of 200 Hz and exported as EDF files for further processing. EEG processing and analysis were performed using NeuroAnalyzer v0.23.9 (accessible at https://neuroanalyzer.org, accessed on 8 January 2024). The processing pipeline included visual inspection of all recordings, processing and analysis. After REM segments were manually identified, we automatically discarded non-REM segments; this resulted in a total of 311 REM segments available for subsequent analysis. Processing included referencing to the contralateral mastoid electrodes (e.g., O2A1 and O1A2), filtering (low-pass FIR filter at 30 Hz) and segmentation into 20-s epochs. Next, for each of the 2091 epochs from all EEG recordings, we calculated the power spectrum (using the Welch periodogram, normalized to dB). We analyzed delta (0.1–4 Hz), theta (4–8 Hz), alpha (8–13 Hz), and beta (14–30 Hz) frequency bands at the F3, F4, C3, C4, O1, and O2 electrode placements (based on International 10/20 System). Band powers were calculated by integrating power spectra using the composite Simpson’s rule. Subsequently, for each subject, the average band powers of REM epochs were computed. All results of the spectral analysis were further analyzed statistically. The EEG processing script can be found at https://codeberg.org/AdamWysokinski/research-data (accessed on 8 January 2024).

DS was scheduled to take place between 2 and 4 weeks following the PSG. Participants were mandated to visit the Department twice, in the evening and the subsequent morning. Participants were permitted to engage in activities throughout the night, provided that they adhered to the protocol.

Actigraphy (Actigraph GENEActive Original, ActivInsights Ltd., Huntingdon, UK) was used to monitor the physical activity (PA) of the study participants. Each participant was equipped with the necessary device during the evening visit to the Department. Records were manually scored by a qualified researcher in accordance with the best current practices set by other authors [42,43]. Actigraphy data were analyzed and presented as a gravity-subtracted sum of vector magnitudes, which represents the intensity of a given activity during a period of time. Additionally, patients were required to declare that they did not fall asleep during DS.

Venous blood samples (9 mL) were collected during the participant’s assessment conducted in the Department at four time points: in the evening of the PSG/DS and on the morning following the procedure.

### 4.3. Molecular Analysis

RNA extraction was performed utilizing the Trizol method (Trizol, Invitrogen) following the manufacturer’s protocol. The quality and quantity of the isolated RNA were assessed using a spectrophotometer (Nanodrop Colibri Microvolume Spectrometer, Titertek Berthold, Germany).

Complementary DNA (cDNA) synthesis was performed using a reverse transcription kit (SuperScript IV First-Strand Synthesis System, Thermo Fisher Scientific Inc., Waltham, MA, USA). The cDNA synthesis reaction was carried out at optimal temperature and time conditions specified by the manufacturer.

Quantitative real-time polymerase chain reaction (qRT-PCR) was employed to analyze the expression levels of the circadian locomotor output cycles kaput (CLOCK), neuronal PAS domain protein 2 (NPAS2), nuclear receptor subfamily 1 group D member 1 (NR1D1), period circadian protein homolog 1 (PER1), cryptochrome circadian regulator 1 (CRY1), and basic helix-loop-helix ARNT-like protein 1 (BMAL1) genes, using beta-actin (ACTB) as a reference gene. Probes were obtained commercially (TaqMan assays). Assays underwent annealing at 60 °C for 60 s. Relative gene expression levels were calculated using the comparative Ct method (2^−ΔΔCt^). All experiments were performed in triplicate to ensure reproducibility.

### 4.4. Statistical Analysis

Data analysis was conducted using Statistica 13.1 PL (StatSoft, Tulsa, OK, USA). A significance level of *p* < 0.05 was established for all statistical tests. The distribution of the data, whether normal or non-normal, was assessed using the Shapiro–Wilk test.

Due to the presence of extreme and outlier values in the expression of the studied genes and taking into account the limited size of the study group, it was decided not to remove these values but to apply a logarithmic transformation.

Descriptive statistics were reported as either mean with standard deviation or median with interquartile range (IQR: first–third quartile) for normally and non-normally distributed data, respectively. Parametric independent variables were assessed using the Student *t*-test, while non-parametric dependent or independent variables were analyzed using the Wilcoxon or Mann–Whitney U test, respectively. Correlations were determined using Spearman’s correlation test.

To identify the key factors influencing PER1 gene expression, we performed a progressive stepwise linear regression analysis.

## Figures and Tables

**Table 1:** Baseline characteristics of the study participants.

N	74
Age (years, median, IQR)	24 (22–26)
BMI (kg/m^2^, mean, SD)	22.9 ± 2.8
Women (*n*, %)	38, 51.4
Smoking (*n*, %)	9, 12.2
Surgical operations (*n*, %)	29, 39.2
Higher education (*n*, %)	34, 45.9
TST in PSG (min, median, IQR)	407.5 (360.5–470.5)
Sleep latencies in PSG (min, median, IQR)	34.5 (22.5–55.5)
Sleep efficiency in PSG (%, mean, SD)	78 ± 10.5
REM duration in PSG (min, mean, SD)	98.7 ± 44.0
NREM duration in PSG (min, mean, SD)	321.8 ± 53.0
Movement in deprivation (median, IQR)	317.5 (252.8–424.9)

Abbreviations: BMI: Body mass index, IQR: Interquartile range, N: Numbers, NREM: Non-rapid eye movement sleep, PSG: Polysomnography, REM: Rapid eye movement sleep, SD: Standard deviation, TST: Total sleep time.

**Table 2:** Comparison of the logarithmic relative expression of selected circadian rhythm genes at four time points (before and after PSG night and before and after sleep deprivation).

	A	B	C	D	*p* (A − B)	*p* (C − D)	*p* (B − D)	*p* (A/B − C/D)
CLOCK	−3.7 (−4.3–(−3.0)), *n* = 57	−3.2 (−3.7–(−2.6)), *n* = 68	−3.1 (−3.7–(−2.5)), *n* = 62	−3.0 (−3.3–(−2.4)), *n* = 71	**0.009**	0.432	**0.027**	**0.033**
BMAL1	−3.9 (−4.4–(−2.9)), *n* = 65	−3.5 (−3.9–(−2.6)), *n* = 70	−3.4 (−4.2–(−2.6)), *n* = 68	−3.0 (−3.5–(−2.6)), *n* = 73	**0.017**	0.183	**0.028**	0.375
PER1	−3.9 (−4.7–(−3.2)), *n* = 53	−2.4 (−3.1–(−2.1)), *n* = 55	−3.5 (−4.0–(−3.2)), *n* = 48	−3.0 (−3.8–(−2.7)), *n* = 53	**<0.001**	0.137	**0.005**	**<0.001**
CRY1	−4.0 (−4.5–(−3.3)), *n* = 59	−3.5 (−3.9–(−2.9)), *n* = 72	−3.5 (−4.3–(−3.0)), *n* = 65	−3.1 (−3.7–(−2.7)), *n* = 71	**0.001**	**0.009**	0.090	0.194
NR1D1	−3.9 (−4.5–(−3.1)), *n* = 59	−3.4 (−3.8–(−2.7)), *n* = 66	−3.4 (−4.2–(−3.0)), *n* = 57	−3.1 (−3.5–(−2.8)), *n* = 66	**<0.001**	**0.034**	0.108	0.120
NPAS2	−4.3 (−4.7–(−3.9)), *n* = 41	−4.0 (−4.3–(−3.5)), *n* = 51	−4.3 (−4.7–(−3.9)), *n* = 48	−3.8 (−4.0–(−3.6)), *n* = 54	**0.015**	**<0.001**	0.434	0.787

A—before PSG, B—after PSG, C—before sleep deprivation, D—after sleep deprivation. Abbreviations: BMAL1: Brain and Muscle ARNT-Like 1, CLOCK: Circadian Locomotor Output Cycles Kaput, CRY1: Cryptochrome Circadian Regulator 1, NPAS2: Neuronal PAS Domain Protein 2, NR1D1: Nuclear Receptor Subfamily 1 Group D Member 1, PER1: Period circadian protein homolog 1, PSG: Polysomnography.

**Table 3:** Correlations between the studied circadian rhythm genes and selected polysomnographic parameters and activity during sleep deprivation.

	B				D
	TST	NREM Dur.	REM Dur.	Latency	Movement
CLOCK	−0.01, 0.942	0.01, 0.906	−0.01, 0.937	−0.06, 0.599	**0.27, 0.022**
BMAL1	−0.07, 0.576	0.06, 0.635	−0.09, 0.453	0.01, 0.916	**0.37, 0.001**
PER1	0.30, **0.024**	0.32, **0.018**	0.08, 0.552	**−0.38, 0.004**	0.04, 0.753
CRY1	−0.05, 0.689	0.01, 0.942	−0.03, 0.794	−0.06, 0.611	**0.28, 0.017**
NR1D1	−0.03, 0.824	0.10, 0.438	−0.05, 0.665	0.10, 0.404	0.24, 0.050
NPAS2	0.07, 0.617	0.10, 0.468	0.00, 0.992	−0.08, 0.578	0.11, 0.419

B—after PSG, D—after sleep deprivation. Abbreviations: BMAL1: Brain and Muscle ARNT-Like 1, CLOCK: Circadian Locomotor Output Cycles Kaput, CRY1: Cryptochrome Circadian Regulator 1, NREM: Non-rapid eye movement sleep, NPAS2: Neuronal PAS Domain Protein 2, NR1D1: Nuclear Receptor Subfamily 1 Group D Member 1, PER1: Period circadian protein homolog 1, PSG: Polysomnography, REM: Rapid eye movement sleep, TST: Total sleep time.

**Table 4:** Association between PER1 logarithmic gene expression measured after a polysomnographic night and the power of brain waves recorded during NREM sleep.

	Alpha	Beta	Theta	Delta
F4A1	28, −0.02, 0.914	28, 0.05, 0.797	28, −0.04, 0.842	28, −0.06, 0.780
F3A2	32, −0.03, 0.891	32, −0.13, 0.470	33, −0.02, 0.903	33, −0.17, 0.346
C4A1	28, 0.01, 0.960	28, 0.12, 0.545	28, −0.04, 0.855	28, 0.00, 0.989
C3A2	32, 0.00, 0.979	32, −0.12, 0.507	33, 0.03, 0.883	33, −0.18, 0.326
O2A1	24, −0.03, 0.878	24, 0.05, 0.818	24, −0.15, 0.475	24, −0.07, 0.734
O1A2	29, −0.38, **0.042**	29, −0.40, **0.033**	29, −0.32, 0.096	29, −0.53, **0.003**

Abbreviations: NREM: Non-rapid eye movement sleep, PER1: Period circadian protein homolog 1. The data are presented as *n*, R, *p*.

## Data Availability

Data are available on request from the authors.
